# Hereditary Tyrosinemia Type 1 in Jordan: A Retrospective Study

**DOI:** 10.1155/2021/3327277

**Published:** 2021-12-02

**Authors:** Noor A. Megdadi, Ahmad K. Almigdad, Mo'men O. Alakil, Shahrazad M. Alqiam, Sumaia G. Rababah, Moshera A. Dwiari

**Affiliations:** ^1^Department of Pediatrics, Royal Medical Services, Jordan; ^2^Department of Orthopedic Surgery, Royal Medical Services, Jordan

## Abstract

**Background:**

Hereditary tyrosinemia type 1 (HT1) is a recessively inherited inborn error of metabolism affecting the final step of tyrosine catabolism. The accumulation of tyrosine toxic metabolites leads to progressive hepatic, renal, and neurological manifestations. Treatment of HT1 consists of tyrosine-restricted diets and nitisinone. The untreated disease progresses into life-threatening liver failure with an increased risk of hepatocellular carcinoma.

**Methods:**

From April 2010 to March 2021, eighteen patients were diagnosed with HT1 in the metabolic department at Queen Rania Al Abdullah Hospital for Children in Jordan. Patients were reviewed retrospectively regarding their clinical features, laboratory data, and sociodemographic history.

**Results:**

The mean age of nine boys and nine girls was 6.03 years (SD ± 3.85). The mean age for symptom onset was 5.61 months (SD ± 6.02). However, the diagnosis was belated from the onset by 10.50 months (±10.42). Nitisinone treatment was delayed from diagnosis around 12.28 months (SD ± 25.36). Most of the patients (66.7%) had acute onset of the disease. Two children (11.1%) died due to hepatic complications. Positive family history was identified in 61.1% of patients, and a similar percentage were born to parents with consanguineous marriage. The most common presentation was abdominal pain, vomiting, and fever. Hepatomegaly and abdominal distention were the most common findings. Six patients' (42.9%) first presentation was rickets.

**Conclusion:**

HT1 diagnosis is usually delayed because it is not part of newborn screening and nonfamiliarity with the clinical features of the disease. Therefore, nationwide newborn screening should be expanded to include HT1.

## 1. Background

Hereditary tyrosinemia type 1 (HT1) is a rare inborn error of metabolism affecting the final step of tyrosine catabolism. HT1 is recessively inherited, where the enzyme fumarylacetoacetate hydrolase (FAH) is deficient [1–3]. The accumulation of tyrosine and its toxic metabolites such as fumarylacetoacetate (FAA), maleylacetoacetate (MAA), succinylacetoacetate (SAA), and succinylacetone (SA) are responsible for the pathogenesis. Consequently, this will lead to progressive hepatic, renal, and neurological manifestations [4, 5].

According to the age of symptom onset, HT1 is classified into acute, where symptoms appear in the first six months of life; subacute if symptoms appear between six and twelve months; and chronic if symptoms appear after one year old [6]. Acute onset is associated with severe features of acute liver failure. Subacute onset disease presented with failure to thrive and developmental delay, melena, jaundice, hepatosplenomegaly, and coagulopathy. Chronic form HT1 has a gradual onset and less severe features. Hypophosphatemic rickets occurs secondary to renal Fanconi syndrome. Neurological symptoms are common and include polyneuropathy and porphyria-like crisis [7, 8]. Hypoglycemia secondary to hyperinsulinism and hypertrophic cardiomyopathy is a less frequent feature [9, 10]. The condition, if not treated, progresses into life-threatening liver failure with an increased risk of hepatocellular carcinoma [11–13].

Treatment of HT1 consists of tyrosine-restricted diets and nitisinone to inhibit the toxic metabolite formation [14–16]. Nitisinone introduction improve survival and reduce the admission rates for the patients [17]. Liver transplantation is the only definitive therapy for the disease [18–20].

In Jordan, studies about HT1 are limited. Therefore, this study was aimed at reviewing all treated tyrosinemia patients' clinical features and outcomes in Jordan and comparing our findings with the literature.

## 2. Methods

In Jordan, all tyrosinemia patients are referred to the metabolic unit at Queen Rania Al Abdullah Hospital for Children (QRHC), an integrated hospital of King Hussein Medical City in Amman, the only unit that treats and follows them. Therefore, this review includes all tyrosinemia-confirmed patients in Jordan. In this retrospective single-center study, we reviewed all patients with HT1 from April 2010 to March 2021.

Patient details and their clinical and laboratory data were extracted from metabolic unit records and the QRHC electronic archiving system. Detailed patients' family history, including consanguinity and siblings with confirmed or suggestive history, was obtained. Presenting clinical features and follow-up history and management were analyzed. Laboratory evaluation includes full blood count, coagulation profile, liver and kidney functions, vitamin D, serum calcium, phosphate, alkaline phosphatase (ALP), plasma tyrosine, phenylalanine, and the plasma and urine succinylacetone. Tumor marker, alpha-fetoprotein and CEA, analyses were done to follow patients regarding the risk of hepatocellular carcinoma, in addition to imaging studies such as abdominal ultrasound and MRI.

### 2.1. Statistical Data Analysis

The mean and standard deviation were used to describe the continuous measures and the frequency and percentages for the categorically measured variables. The multiple response dichotomy analysis was applied to describe the children's presenting signs and symptoms.

## 3. Results

Table 1 displays the descriptive analysis for the eighteen children (nine boys and nine girls) with tyrosinemia type 1. Most of the patients (66.7%) had acute onset of the disease. Two children (11.1%) died due to hepatic complications. The mean age of patients at the time of the study was 6.03 years (SD ± 3.85). The mean age for symptom onset was 5.61 months (SD ± 6.02), but the mean age of diagnosis was later 10.50 months (±10.42). Nitisinone treatment was delayed from diagnosis around 12.28 months (SD ± 25.36).

The children's family history (Table 2) showed a positive history of HT1 in 61.1% of patients. Additionally, 61.1% of patients were born to parents with consanguineous marriage. Half of the patients had affected siblings with HT1 disease. Cirrhosis in relatives and unexplained sibling death were frequent too.

**Figure 1:**
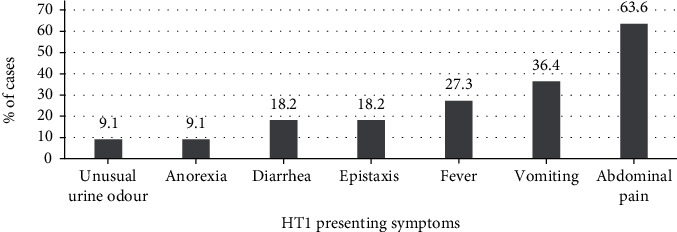
Presenting symptoms of the HT1.

**Figure 2:**
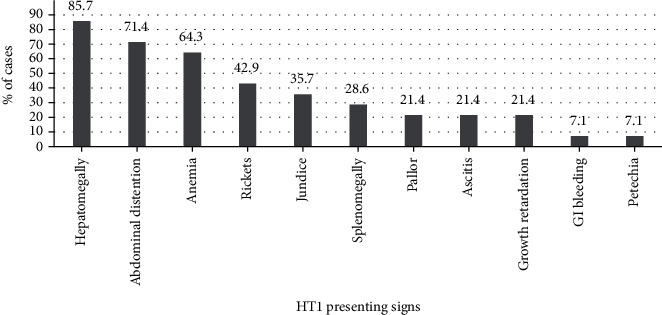
Presenting signs of HT1.

Figures 1 and 2 showed the presenting symptoms and signs of HT1 at diagnosis. The most common presentation was abdominal pain, vomiting, and fever. On the other hand, hepatomegaly and abdominal distention were the most common findings of those patients. Six patients' (42.9%) first presentation was rickets.

**Figure 3:**
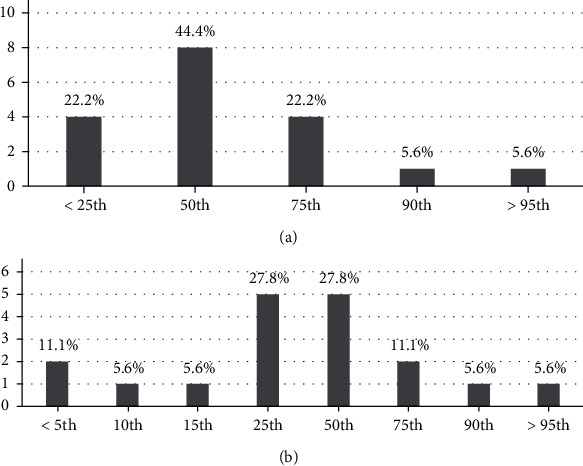
(a) Weight percentile. (b) Height percentile.

Figures 3(a) and 3(b) demonstrate weight and height percentile. Although there is a systemic effect of HT1, growth retardation was found in only 21.4% of patients.

Table 3 displays the laboratory findings and workup at presenting time. Complete blood count revealed anemia in 44.4% of patients and thrombocytopenia and thrombocytosis in 38.9% and 5.6%, respectively. An abnormal coagulation profile was present in the majority of patients. Elevated aspartate aminotransferase and gamma glutamyl transferase are predominant in liver function tests. In contrast, a low albumin level was noticed in a third of patients at presentation. Total and direct bilirubin was elevated in 44.4% of patients. Alkaline phosphatase was markedly elevated with a mean of 897.7 IU/L (±829.1) in more than half of the patients. The tumor marker's alpha-fetoprotein mean level was 47807.56 ng/mL (±100578.37), and this elevation was found in 55.6%. Plasma levels of succinylacetone, tyrosine, and methionine were elevated in 33.3%, 77.8%, and 38.9%, respectively. 27.8% of patients had low phenylalanine levels.

Most hospitalizations were for diagnostic workup or secondary to electrolyte disturbance. Nevertheless, gastrointestinal bleeding was responsible for frequent admissions in two patients.  Table 4 shows the details of 18 patients.

## 4. Discussion

The worldwide birth incidence of HT1 approximates 1 in 100,000 [16], where the highest incidence is in Quebec in Canada, which was up to 1 in 16,000 [21]. However, the exact incidence of HT1 is unknown in Jordan and secondary to high consanguineous marriage; the incidence is expected to be high. In this study, parents' consanguinity was found in 11 out of 18 patients (61.1%). Nevertheless, HT1 is not a part of the newborn screening program in Jordan. Nationwide newborn screening is limited to phenylketonuria, congenital hypothyroidism, and G6PD deficiency. Four patients were diagnosed by selective screening due to positive family history. Many confirmed HT1 patients had a positive history of unexplained sibling death or a positive family history of hepatic failure and cirrhosis. Therefore, the true incidence is expected to be higher.

Because of lack of screening and insidious onset of the chronic form of HT1, there was a diagnosis delay around five months after the onset of symptoms. Delayed treatment is associated with increased complications and a higher risk for hepatocellular carcinoma. Our review includes patients since 2010 while the nitisinone was introduced to the metabolic unit in February 2014. Therefore, a significant number did not receive nitisinone and were treated by a tyrosine-/phenylalanine-restricted diet. Even though nitisinone treatment was introduced in our metabolic unit, there is still a delay in treatment, either due to a delay in diagnosis or the need to refer patients from different medical sectors in Jordan to our unit. The mean delay between diagnosis and initiation of nitisinone treatment was 12.28 months (±25.36). Compliance problems with dietary restriction were more notable than compliance with nitisinone treatment.

The acute form of HT1 has severe clinical findings compared to the subacute and chronic form, explaining earlier diagnosis. Abdominal pain, vomiting, and fever were the most predominant symptoms. Abdominal distention secondary to hepatosplenomegaly was the most common finding in clinical exams. Though there is a systemic effect of the disease, growth retardation was found in only three patients.

Mild anemia was found in 44.4% of patients. Although the coagulation profile showed prolonged INR in 77.7% of patients, gastrointestinal bleeding and petechial occurred in only two patients and were responsible for frequent hospitalization.

Elevation of liver enzymes at presentation was the most striking laboratory finding, and AST and GGT were most markedly affected. Elevated bilirubin level was found in eight patients, while clinical jaundice was seen in five patients.

Alpha-fetoprotein is used as a tumor marker to follow the patients for hepatocellular carcinoma in conjunction with serial liver ultrasound. AFP was significantly raised in 55.6% of patients with a mean value of 100578.37 ng/mL. HCC risk increased in the chronic form of HT1 and those who received nitisinone treatment after the age of two years. HCC should be expected in a case of persistently elevated AFP or slow level decreasing without normalization and in those with a secondary increase in AFP. HCC was reported in one female patient in our review; she received nitisinone at the age of four years.

Regarding plasma level, tyrosine was elevated in around half of patients and Succinylacetone in third. Therefore, their plasma level is not suitable as a screening test for HT1.

Renal tubular dysfunction is common in HT1 and caused by the nephrotoxic effect of tyrosine metabolites. Hypophosphatemia and low vitamin D were significant and were found in more than two-thirds of patients. Consequently, hypophosphatemic rickets was diagnosed in six patients; one patient underwent corrective osteotomy due to severe genu varum deformity.

Although cardiomyopathy is a frequent finding of HT1, none of our patients was reported to develop it. Nitisinone reduces cardiomyopathy complications of the disease by reducing the circulating metabolites [10]. The intellectual function of HT1 patients was evaluated in many studies. Bendadi et al. performed a cross-sectional study to establish cognitive functioning in children with HT1 compared with their unaffected siblings. Bendadi et al. found that patients with HT1 treated with nitisinone are at risk for impaired cognitive function despite a protein-restricted diet [22]. In our review, no neurological findings were reported, and intellectual function was not assessed.

Comparative studies are limited in Jordan; therefore, we utilize regional and international ones. Zeybek et al. reviewed thirty-eight patients over twenty years in Turkey [23]. Similarly, no screening test in Turkey results in a delay in diagnosing and starting nitisinone treatment. Dietary compliance problems were frequent in the Zeybek review. Eleven patients underwent cognitive evaluation, and they were found to have a lower intellectual function even with nitisinone treatment. Six patients had liver transplantation despite nitisinone treatment: three due to suspected HCC, two for noncompliance to diet, and one for both.

Nakamura et al. conducted a nationwide survey of hereditary tyrosinemia disease in Japan. The incidence in Japan of HT1 is exceptional. In their survey, even patients treated early with nitisinone may develop liver cancer [24].

Äärelä *et al*. reviewed twenty-two children with HT1 in Finland between 1978 and 2019. The incidence was 1 in 90,102. At diagnosis, the median age was five months; four patients were detected through screening and received early treatment. Seven patients underwent liver transplantation. Liver values normalized in thirty-one months, and other laboratory values normalized except thrombocytopenia within eighteen months. On the other hand, imaging findings normalized in 3–56 months, excluding five patients with liver or splenic abnormalities [25].

In another study from Mexico, González et al. reviewed twenty patients. Patients had classical features of hepatorenal tyrosinemia, but the presentation is later compared to other studies. The mean age at onset of clinical symptoms was 10.18 months (range 0.3–60 months), and the mean age at diagnosis was 19.48 months (1.3–60.9 months). Four underwent hepatic transplantation because of advanced cirrhosis and hepatic nodules [26].

## 5. Conclusion

Diagnosis of HT1 is usually delayed in Jordan because it is not part of newborn screening and because of the physician's nonfamiliarity with the clinical features of the broad-spectrum disease. Consanguineous marriage is common in Jordan. Consequently, an inborn error of metabolism diseases is expected to be high. Therefore, nationwide newborn screening should be expanded to include HT1.

## Figures and Tables

**Table 1:** Descriptive analysis of the HT1 sociodemographic characteristics.

	Frequency	Percentage
Gender		
Female	9	50
Male	9	50
Classification of HT1 based on the age of symptom onset		
Acute (<6 months)	12	66.7
Subacute (6-12 months)	5	27.8
Chronic (>12 months)	1	5.6
Survival		
Alive	16	88.9
Died	2	11.1
Age (years) at the study time, mean (SD)	6.03 (3.85)	
Age (months) at symptom onset, mean (SD)	5.61 (6.02)	
Age (months) at diagnosis time, mean (SD)	10.50 (10.42)	
Age (months) at the commencement of NTCB therapy, mean (SD)	20.33 (25.97)	
The interval (months) between diagnosis and initiation of NTCB therapy, mean (SD)	12.28 (25.36)	

**Table 2:** Descriptive analysis of the HT1 diagnosed children's family history, *N* = 18.

	Frequency	Percentage
Parents consanguinity	11	61.1
The degree of kinship between the parents		
First cousins	9	50
Second cousins	2	11.1
Not related	7	38.9
Family history of HT1	11	61.1
Affected siblings with HT1	9	50
History of unexplained sibling death	4	22.2
Any close relatives with cirrhosis	2	11.1
Any far relatives with cirrhosis	3	16.7
Hepatocellular carcinoma in relative	1	5.6

**Table 3:** Descriptive analysis of the HT1 diagnosed children's presenting laboratory results.

	Mean (SD)	Frequency	Percentage
Full blood count (CBC)			
Low serum hemoglobin level	11.33 g/d (2.00)	8	44.4
Serum platelet	217.00 cells/mm^3^ (120.31)		
Thrombocytopenia		7	38.9
Thrombocytosis		1	5.6
Blood coagulation studies			
Prolonged PT	20.78 seconds (10.53)	14	77.8
High INR	1.67 (1.03)	14	77.8
Prolonged aPTT time (seconds)	36.72 seconds (8.64)	4	22.2
Kidney function tests			
Low serum creatinine level	0.250 mg/dL (0.104)	5	27.8
Serum urea (mg/dL) level	18 mg/dL (4.68)		
Low		4	22.2
High		1	5.6
Liver function tests			
High AST level	135.67 IU/L (313.52)	15	83.3
High ALT level	84.06 IU/L (223.30)	4	22.2
High GGT level	64.39 IU/L (43.47)	13	72.2
High total protein level	6.44 g/dL (0.922)	2	11.1
Low serum albumin level	4.00 g/dL (0.60)	6	33.3
High serum total bilirubin level	2.78 mg/dl (4.85)	8	44.4
High serum direct bilirubin level	1.33 mg/dl (2.47)	8	44.4
Bone profile			
Serum calcium level (normal)	9.50 mg/dL (0.62)	18	100
Serum phosphate level	3.67 mg/dL (1.53)		
Low		13	72.2
High		1	5.6
Low serum vitamin D level	18.39 ng/mL (7.28)	12	66.7
High ALP level	897.7 IU/L (829.1)	10	55.6
Tumor marker			
High AFP level	47807.56 ng/mL (100578.37)	10	55.6
High CEA level	2.50 ng/mL (1.43)	7	38.9
Plasma studies			
High plasma succinylacetone	2.28 *μ*mol/L (2.72)	6	33.3
High tyrosine level	295.39 *μ*mol/L (201.74)	14	47.72
Low phenylalanine level	47.72 *μ*mol/L (33.74)	5	27.8
High methionine level	58.22 *μ*mol/L (36.18)	7	38.9

**Table 4:** Summary of the patients' data, *N* = 18.

Case number	Gender	Age (years)	Age at diagnosis (months)	Age of symptom onset (months)	Time between onset and diagnosis (months)	Age of NTBC initiation (months)	Interval between diagnosis and NTBC treatment (months)	Growth retardation	Number of hospitalizations	Cause of admission	Alive/dead
1	Male	1	4	4	0	4	0	No	0		Alive
2	Female	4.50	6	5	1	7	2	No	0		Alive
3	Male	3.89	31	3	28	29	26	No	0		Alive
4	Male	7.13	12	9	3	15	6	No	3	Abdominal pain	Alive
5	Female	5.17	1	0	1	2	2	No	1	Chest infection	Alive
6	Female	3.71	7	7	0	7	0	No	1	Chest infection	Alive
7	Male	2.19	2	2	0	2	0	No	1	Electrolyte imbalance	Alive
8	Female	7.47	12	6	6	18	12	Yes	1	Electrolyte imbalance	Alive
9	Male	0.67	2	0	2	2	2	Yes	2	Electrolyte imbalance	Alive
10	Female	6.93	6	3	3	6	3	No	1	Encephalopathy	Alive
11	Male	1.59	1	1	0	1	0	No	1	Fever	Alive
12	Female	14.42	3	3	0	108	108	No	1	For diagnostic workup	Alive
13	Male	7.41	12	3	9	12	9	No	0	For diagnostic workup	Alive
14	Female	4.27	12	12	0	12	0	No	1	For diagnostic workup	Alive
15	Male	10.47	18	9	9	36	27	No	8	GI bleedings	Alive
16	Male	6.30	19	5	14	19	0	No	2	Orthopedic surgery (genu varus)	Alive
17	Female	12^∗^	3	3	0	48	14	No	10	GI bleedings	Died
18	Female	5^∗^	38	26	12	38	10	Yes	2	Hepatic failure	Died

^∗^Passed (patient number 17 died secondary to HCC; patient number 18 died secondary to hepatic failure).

## Data Availability

The data used to support the study can be available upon request.
